# Health Tract, No. 90

**Published:** 1862-07

**Authors:** 


HEALTH TRACT, NO. 90.

### SOLDIERS REMEMBERED

If you write to a soldier, friend, or relative in the army, using a common envelope and a sheet of foolscap-paper, you may also add, without exceeding the weight for which a three-cent postage-stamp will pay, as much tea as a teaspoon will take up twice, or as much black or cayenne pepper, such as is obtained from a good drug-store under the name of "Capsicum," as you can take up at once with a common teaspoon, and the smaller envelope of thin paper to hold either. Chewing the tea, a pinch at a time, every hour or half-hour, while keeping guard, or under circumstances of great thirst, or of excessive weariness or sleepiness, will enliven, will modify thirst, will invigorate, or will waken up to a grateful extent, considering the amount of tea used, and its perfect safety from ulterior ill results, such as follow the use of alcoholic drinks. But a heaping teaspoonful of genuine "Capsicum" is worth ten-fold its weight of tea-leaves, especially in summer, in many ways; for example, a single quarter of a pinch will save a man's life—that quarter of a pinch being put in a sleepy sentinel's eye. If it don't waken him up, and every body else within an Indian yell's distance, then it is not a prime article of capsicum. A single pinch in a glass of flat or warmish water will nullify these qualities, and besides satisfying thirst, will invigorate and effectually prevent that uncomfortable sensation arising from having drank largely of water. A good pinch, eaten at each meal, or whenever a cup of coffee or tea or water is swallowed, will always invigorate digestion, aids to prevent acidity, and is, besides, a great antagonist of the diarrhea, dysentery, flux, and "looseness," which are the great scourges of all armies. A level teaspoon of capsicum daily, taken in eating or drinking, or both, or if taken a pinch at a time during the day or night, would do more real good, and that without any ill result, than ten times the cost in rum and quinine, as a preventive against chills and fevers. Liquor and quinine initiate the soldier into intemperate habits; they will wake up a love, a craving, a slavery to strong drink, which pepper and water will never do. The latter invigorates like food, the former merely excites, then leaves weaker than before. A pinch of capsicum, which is simply pure cayenne pepper, will do a great deal more toward warming up a soldier, toward invigorating him, toward keeping him vigilant on guard, and toward modifying thirst or fatigue, than the best glass of grog ever swallowed. Capsicum goes farther, and is more efficient for all purposes, than black pepper; if by express or privately, send half a pound at a time, in a tin box. If you have nothing else to send in your letters, send a few pins, or a needle and some thread. Many have seen the time when a string or a pin would have been worth ten times its ordinary value. Write often to the soldier. Write long letters. Give all the news you can think of. Let every line be full of love; of kind, affectionate interest and encouragement. Be sure to inculcate a generous magnanimity toward those who oppose, *so as to have as few obstacles as possible to a cordial coming together again*, when that good time comes, as it certainly will, before long. We are all brethren, presently estranged, sons of the same sires, and, taking an enlarged view, the aggregate character is pretty well balanced.

### PIANO-FORTE.

H. WORCESTER offers for sale a large assortment of choice PIANO-FORTES, 6, 6¾, 7, and 7¼ octaves, in elegant rosewood cases — prices, from $225 to $700—all of which are manufactured under his own supervision, and are for sale on reasonable terms, in Fourteenth street, corner Third avenue, New-York.

By devoting his personal attention to the touch and tone of his instruments, which have hitherto been considered unrivaled, he will endeavor to maintain their previous reputation, and respectfully solicits an examination from the profession, amateurs, and the public.

"There is one of these charming instruments near us, made by WORCESTER, which, for its power of action, for the richness, clearness, and SWEETNESS of its tone, is the more remarkable from the fact of its having been in use TWELVE YEARS. This manufactory well maintains its old reputation for the DURABILITY of its instruments. Being intended for *use* rather than for mere show, they are made in the most SUBSTANTIAL MANNER ; and, for this reason, we specially command them to our Western and Southern readers. The extra time and labor expended on many pianos, to make them sell well, is put upon WORCESTER'S instruments to render them MORE LASTING, and to defer the necessity of repairs for a longer period. The frailty with which many instruments for Southern use are made, and the unskillfulness of most persons at a distance from large cities, who profess to repair and tune them, very often, as families know by experience, soon render them almost unfit for use. For these reasons, the WORCESTER piano is not only afforded at LESS COST now, but is subject to LESS RISK and expenditure hereafter, in keeping it in tune."—*April Journal of Health.*

"We have, in many ways, and at many times, expressed our opinion strongly in praise of American mechanical art; and a recent visit to the extensive piano manufactory of HORATIO WORCESTER, has strengthened that opinion; and we are well convinced that in the construction of these beautiful instruments, we Americans are rivaling, if not excelling, Europeans. The Philharmonic Society, the most scientific musical association in this country, use WORCESTER'S pianos at their grand concerts. We have had one in constant use for the last fourteen years, and find no diminution in clearness or sweetness of tone. They are said to stand every climate, and are daily being exported to the West-Indies, Canada, the far West, and South. The factory has been newly fitted up in the neatest manner, and ladies visiting it will find every facility for making their selections."—*Hon. Erastus Brooks, in New-York Express.*

"WORCESTER'S instruments, for perfection of finish and beauty of tone, seem to us unsurpassed. He has lately introduced an improvement, in respect to the sounding-board, that may make a mark upon the future of the piano manufacture. They who wish to see it in operation will find a kind and courteous reception at the rooms in Fourteenth street. Mr. WORCESTER has the latest improvements in his art, and his instruments are of the newest and best make; yet, in one respect, he is a very old-fashioned man, for he speaks the truth after the ancient way of our fathers, and if any friends should send him an order by mail, they may rely upon it that it will be executed promptly and faithfully, at the fairest market rates."— *Christian Inquirer.*

"Why WORCESTER'S instruments maintain their acknowledged superiority, was lately explained by one of the very best piano workmen in the city, not in Mr. WORCESTER'S employ. 'There is no shop known to me where such extraordinary pains are taken to make every part of the instrument as perfect as possible, as in the old and extensive establishment of HORATIO WORCESTER."—*New-York Journal.*

